# Retraction Note: Down-regulation of kappa opioid receptor promotes ESCC proliferation, invasion and metastasis via the PDK1-AKT signaling pathway

**DOI:** 10.1186/s12964-023-01205-1

**Published:** 2023-06-27

**Authors:** Han-Ming Huang, Xin-Hua He, Xiao-Yu Huang, Guo-Yun Wang, Qiao-Xi Xia, Ze-Peng Du, Yong-Fa Zhang

**Affiliations:** 1 Department of Anesthesiology, Second Affiliated Hospital of Shantou University Medical College, Shantou, 515041 People’s Republic of China; 2 Department of Physiology, Shantou University Medical College, Shantou, 515041 People’s Republic of China; 3 Shenzhen Qianhai Shekou Free Trade Zone Hospital, Shenzhen, 518067 People’s Republic of China; 4 Central Laboratory, Shantou Central Hospital, Shantou, 515041 People’s Republic of China; 5 Department of Pathology, Shantou Central Hospital, Shantou, 515041 People’s Republic of China

**Retraction Note: Cell Commun Signal 20, 35 (2022)**

**https://doi.org/10.1186/s12964-022-00833-3**

The Editor has retracted this article on request from the authors. After publication, the authors became concerned that the KYSE180 cell line used in this study was contaminated. In addition, image overlap has been found in Fig. 2F. The authors stated overlapping images for cell line KYSE30 were wrongly used to present data for cell line KYSE180. Readers should disregard this figure.

Authors Han-Ming Huang, Xin-Hua He, Guo-Yun Wang, Ze-Peng Du, Qiao-Xi Xia and Yong-Fa Zhang agree to this retraction. Author Xiao-Yu Huang has not responded to correspondence from the publisher or editor about this retraction.

